# Does lactate clearance prognosticates outcomes in ECMO therapy: a retrospective observational study

**DOI:** 10.1186/s12871-018-0618-1

**Published:** 2018-10-24

**Authors:** İbrahim Mungan, Dilek Kazancı, Şerife Bektaş, Derya Ademoglu, Sema Turan

**Affiliations:** Department of Intensive Care Unit, Turkey Advanced Speciality Education and Research Hospital, Altındag, Ankara, Turkey

**Keywords:** Extracorporeal membrane oxygenation, Lactate, Clearance

## Abstract

**Background:**

ECMO support is a final treatment modality for patients in the refractory cardiogenic arrest and postcardiotomy cardiogenic shock with an utmost importance. Eventhough it is linked to high mortality, its usage gains popularity worldwide. We assessed the fluctuation of lactate levels and the clearance of lactate during the ECMO therapy and its prognostic role on mortality.

**Methods:**

Data were gathered on all patients receiving ECMO therapy longer than 48 h between January 2015 and December 2017 retrospectively. Blood lactate had been recorded before ECMO implantation and at specific time points during ECMO support as a routine procedure. In this study, the Lactate clearance at specific time points (Lactate clearance-1) and the duration that lactate cleared more than 10% of the initial lactate level (Lactate clearance-2) was measured. Statistical analysis included Mann Whitney U-test and ROC-curves to predict 30-day mortality.

**Results:**

Fourty-eight patients underwent ECMO therapy for refractory cardiogenic shock resulting in 70.8% mortality. The lactate levels before and after ECMO therapy as well as the dynamic changes were significantly correlated with mortality variable. With AUC calculation, LC-2 has a strong discrimination (AUC = 0.97) on 30-day survivors and nonsurvivors. LAE-LBE (AUC = 0.785), L48-LBE (AUC = 0.706) showed moderate predictive power on 30-day mortality.

**Conclusions:**

Changes in lactate levels after ECMO implantation is an important tool to assess effective circulatory support and it is found superior to single lactate measurements as a prognostic sign of mortality in our study. Based on our results, an early insertion of ECMO before lactate gets high was suggested. Serial changes on lactate levels and calculation of its clearance may be superior to single lactate on both effective circulatory support and as prognostic prediction. LC-2 showed a strong discrimination on 30-day mortality.

## Background

During cardiogenic shock and cardiac arrest, oxygen supply and blood perfusion are critically reduced; these deteriorations can be corrected by extracorporeal mechanical life support in principle [1]. Extracorporeal membrane oxygenation (ECMO) is one of the methods used to provide temporary mechanical support of the cardiac and/or pulmonary function. It has two types of usage and Veno-arterial-ECMO (V-A ECMO) decreases preload and increases the aortic flow and end-organ perfusion. In this way, ECMO can safeguard the myocardium which ensures better survival [2]. The other type [Veno-venous ECMO (V-V ECMO)] is mainly used with lung-protective ventilation for the treatment of acute respiratory distress syndrome and respiratory failure without cardiac failure [1]. Cardiac surgery–as in post-cardiotomy low cardiac output syndrome-, acute myocardial infarction, decompensated cardiomyopathy, Takotsubo syndrome, or acute myocarditis may be complicated by severe myocardial dysfunction ensuing in cardiogenic shock and consequently requiring ECMO as a treatment option [3].

Lactate is a metabolic product of anaerobic glycolysis and may express inadequate oxygen delivery. It is also suggested to be an indicator of tissue perfusion affected not only by macrocirculation but also by microcirculation [4]. After cardiac arrest or cardiac surgery, discordance between O_2_ demand and supply causes an increase in lactate level, which reflects the imbalance between production and clearance of lactate. This increment in lactate level has been associated with poor prognosis and mortality [5].

Lactate is mainly cleared by the liver, skeletal and cardiac myocytes, and proximal tubule kidney cells. It is claimed that the cause of hyperlactatemia in patients who get stabilized via ECMO support is the decrease in lactate clearance rather than increased production of lactate as in sepsis without hemodynamic instability [6].

Even recent reports concluded that serial lactate measurements and lactate clearance are more reliable for risk stratification than absolute levels of lactate [7]. In this study, we hypothesized that the lactate clearance in different time points (LC-1) or the duration to clear more than 10% of original lactate (LC-2) may show a better correlation with clinical outcomes on ECMO support after cardiac surgery or cardiogenic shock.

## Methods

This retrospective analysis was performed with all consecutive patients receiving a V-A ECMO for either refractory cardiogenic shock or cardiac arrest between January 2015 and December 2017 in a tertiary state hospital. In this study, only patients undergoing at least 48 h of ECMO therapy were included to allow assessment of serial lactate measurements during ECMO support. Patients undergoing V-V ECMO support and patients requiring ECMO support for respiratory failure and patients dead before 48 h were excluded from the analysis. An extra formal consent other than the patients had given prior to the admission to intensive care unit (ICU) as a routine procedure, was not required for the current study because it was a case–control medical record review. Since our study was in the category of non-interventional clinical research with its retrospective structure, no ethics committee approval was applied. This situation is in line with the National Code of Clinical Research which was published on 13th April 2013 [8].

This study adhered to the principles in accordance with the Helsinki Declaration of 1975, as revised in 2008.

### ECMO indication

Intraoperative ECMO support indications were either failure to wean cardiopulmonary bypass due to circulatory instability or hemodynamic impairment after weaning. Postoperative ECMO support indications were refractory postcardiotomy cardiogenic shock and decompensated cardiomyopathy in spite of optimized inotropic therapy and supportive measures (e.g. intra-aortic balloon pump). This proposed situation was defined by systolic arterial hypotension (< 80 mmHg), signs of end-organ failure (urine output < 0.5 ml/h/kg or need for dialysis as renal failure; persistent elevated lactate levels > 3 mmol/l, central venous oxygen saturation (ScvO_2_) < 50% as respiratory failure) and low cardiac output (cardiac index < 1.8 l/min/m^2^ Body surface area). In patients presenting cardiogenic shock as in cardiac arrest, without previous cardiac surgery, identical clinical criteria were applied for postoperative ECMO indications. In patients under resuscitation, the decision to implant ECMO was made by the participating cardiac surgeon. The improvement in respiratory functions, decreasing trend of lactate levels and improved hemodynamics were the criteria for weaning off ECMO support.

### Data acquisition

All clinical variables of patients, necessitating ECMO support were retrospectively recorded in our institutional database. Renal failure was defined as the presence of oliguria (< 0.5 ml/kg/h), the optimisation of fluid loading and a doubling of post-ECMO creatinine values with the need for renal replacement therapy (hemodialysis or filtration). This definition was corresponding to Stages 2 and 3 according to the Kidney Disease: Improving Global Outcomes (KDIGO) definition of acute kidney injury (AKI) [9]. The neurological complication was defined as the presence of clinical or radiological evidence for a neurological deficit or defect, e.g. stroke, severe cerebral bleeding or severe cerebral oedema. Mortality as a variable in this study was described as death from any cause occurring within 30-days after ECMO implantation.

Blood lactate values were measured by arterial blood gas analysis on ICU hourly in the first hours after ECMO implantation until stable conditions were achieved. Later on, lactate values were determined in a two-hour interval. There are so many conflicts in the literature defining lactate clearance to assess its predictive power and we chose two defining models as lactate clearance level. Both of them based on the lactate value before ECMO implantation. Lactate clearance-1 (LC-1) was calculated by the following equation: Lactate clearance (time point) = lactate value before ECMO implantation – lactate value at the specific time point. The other definition of the lactate clearance [lactate clearance-2 (LC-2)] was duration between ECMO implantation and time point that lactate cleared more than 10% of the initial lactate level.

### Statistical analysis

Statistical analysis was performed using SPSS version 20.0 for Windows (SPSS Inc., Chicago, IL, USA) and MedCalc 15.8 software (MedCalc, Ostend, Belgium). Data were analyzed, and the continuous variables were reported as mean ± standard deviation (SD), and nominal variables were reported as total number and percentages.

Variables were first evaluated by One-Sample Kolmogorov-Smirnov test as a normality test to choose the type of statistical tests –parametric or non-parametric test–, and the results showed that asymp. Sig. (2-tailed) levels ≤0.05 so we decided to use non-parametric tests. For statistical analysis, correlations between variables were evaluated for significance by using the Spearman’s rho test. Categorical variables were evaluated by the Mann-Whitney U test of contingency. In all analyses, a ‘*p*’ value of less than 0.05 was considered statistically significant.

Apart from this, we established a receiver operating characteristic (ROC) curve to evaluate the ability of LC-1 and LC-2 to predict 30-day mortality. In this analysis, ROC-Area Under Curve (AUC) was calculated to quantify the accuracy of the predictive model. AUC value > 0.75 was appraised as satisfactory, AUC value > 0.8 was appraised as well, and AUC value > 0.9 was appraised as very good.

## Results

**Fig. 1:**
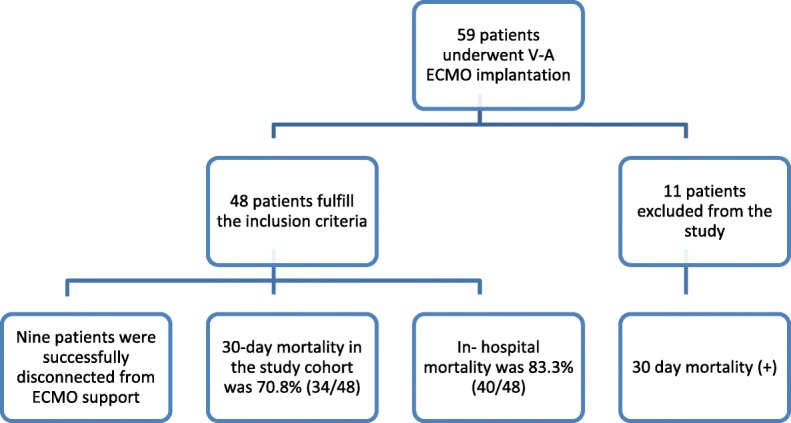
The flowchart describing the initial and study population

From January 2015 to December 2017, 59 patients underwent V-A ECMO implantation in our hospital for either post-cardiotomy refractory cardiogenic shock, decompensated cardiomyopathy or cardiac arrest. Only 81.3% (48/59) of these patients had at least 48 h of ECMO support and fulfill the inclusion criteria of our study (Fig. 1). Nine patients were successfully disconnected from ECMO support and seven patients were switched to the left ventricular assist device (LVAD). Thirty-day mortality in the study cohort was 70.8% (34/48), while 18.6% (11/59) of patients with ECMO support less than 48 h died, resulting in a cumulative 30-day mortality of 76.2% (45/59) in this time period. In-hospital mortality for the study population cohort was 83.3% (40/48) with cardiac death (31/48; 64.5%) being the most frequent cause of death. Other causes of death were sepsis (3/48; 6.3%), mesenteric ischemia (1/48; 2.1%), cerebral death (2/48; 4.2%), untreatable bleeding (2/48; 4.2%) and other causes not assigned to the categories mentioned before (1/48; 2.1%). Demographic data and clinical scenario before ECMO implantation of the included patients are shown in Table 1.

**Table 1:** Demographic data and clinical scenario before ECMO implantation of patients undergoing ECMO for cardiogenic shock

	All (*n* = 48)	Survivors (*n* = 14)	Non-survivors (*n* = 34)	*p*-value^*^
Age (Years)	54 ± 13	47 ± 11	57 ± 12	0.006
Gender (Male)	31 (64.6%)	10 (71.4%)	21 (61.8%)	0.535
ECMO support indications				
Postcardiotomy cardiogenic shock	26 (54.2%)	8 (57.1%)	18 (52.9%)	0.823
Cardiac arrest	15 (31.3%)	4 (28.6%)	11 (32.4%)	
Decompensated cardiomyopathy	7 (14.6%)	2 (14.3%)	5 (14.7%)	

Subgroups (The survivors versus Non-the survivors) refer to 30-day mortality. *p*-values calculated for comparison of the survivors versus non-the survivors by statistical analysis

Data are mean ± SD or *n* (%)

^*^Determined by Mann-Whitney U test or Spearman’s rho test

*Abbreviations*: *ECMO* extracorporeal membrane oxygenation

The survivors regarding 30-day mortality were younger. Other demographic variables were similar and not significant statistically. The clinical situation (e.g.decompensated cardiomyopathy, etc.) before ECMO implantation had no impact on 30-day mortality, length of stay (LOS) in the hospital or in the ICU. ECMO data of the complete patient cohort with ECMO support more than 48 h as well as complications and outcomes after ECMO support are summarized in Table 2.

**Table 2:** Complications and outcomes after ECMO therapy

	All (*n* = 48)	Survivors (*n* = 14)	Non-survivors (*n* = 34)	*p*-value^*^
Postoperative ECMO	33 (68.7%)	10 (71.4%)	23 (67.6%)	0.802
ECMO during CPR	15 (31.3%)	4 (28.6%)	11 (32.4%)	
Central cannulation	13 (27.0%)	3 (21.4%)	10 (29.4%)	0.581
ECMO implantation at ICU	30 (62.5%)	8 (57.1%)	22 (64.7%)	0.632
ECMO related complications				
Severe Bleeding	4 (8.3%)	1 (7.1%)	3 (8.8%)	0.730
Limb ischemia	4 (8.3%)	1 (7.1%)	3 (8.8%)	
Intracardiac thrombus	1 (2.1%)	1 (7.1%)	0 (0%)	
Renal failure with dialysis	30 (62.5%)	6 (42.9%)	24 (70.6%)	0.074
Neurological complication	15 (31.3%)	5 (35.7%)	10 (29.4%)	0.676
ECMO duration (days)	8.5 ± 9.4	14.21 ± 15.0	6.2 ± 4.1	0.131
LOS in-hospital (days)	16.3 ± 18.5	38.79 ± 20.3	7.0 ± 4.9	< 0.001
LOS in-ICU (days)	11.1 ± 11.8	21.5 ± 17.0	6.9 ± 4.5	< 0.001
Successful ECMO weaning	9 (18%)	9 (64.3%)	0 (0%)	< 0.001
LVAD	7 (14.6%)	1 (7.1%)	6 (17.6%)	0.359
30-day mortality	34 (70.8%)			
In-hospital mortality	40 (83.3%)			

Subgroups (The survivors versus Non-the survivors) refer to 30-day mortality. *p*-values calculated for comparison of the survivors versus non-the survivors by statistical analysis

Data are mean ± SD or *n* (%)

^*^Determined by Mann-Whitney U test or Spearman’s rho test

*Abbreviations*: *ECMO* extracorporeal membrane oxygenation, *CPR* cardiopulmonary resuscitation, ICU intensive care unit, *LOS* length of stay, *LVAD* left ventricular assist device

ECMO duration is longer for the survivors compared to non-the survivors yet this was expected as therapy continues in the survivor group. ECMO implantations were done as extracorporeal cardiopulmonary resuscitation (eCPR) refractory to conventional CPR in 31.3% (*n* = 15) of the cases. ECMO implantations were performed in the ICU in 62.5% (*n* = 30) of the study cases. ECMO implantation in the ICU and eCPR usage as a variable had no significant relation statistically with mortality. The most common ECMO-related complications were severe bleeding (8.3%) and limb ischemia (8.3%), particularly in peripheral ECMO implantation.

If patients were hemodynamically stable and adequately oxygenated, they were considered for ECMO weaning when the flow rate was 1 l/min/m^2^ for 4 h. Survival of more than 12 h after discontinuation of ECMO support was defining successful ECMO weaning and accomplished only in nine patients (18.8%).

In the survivor group of patients with ECMO support, the LOS in-hospital (38.79 ± 20.3 days vs 7.0 ± 4.9 days) and LOS in ICU (21.5 ± 17.0 days vs 6.9 ± 4.5 days) were significantly longer. In this study, 62.5% (*n* = 30) of ECMO support patients developed acute renal failure and 31.3% of them had neurological complications.

### Blood lactate

**Fig. 2:**
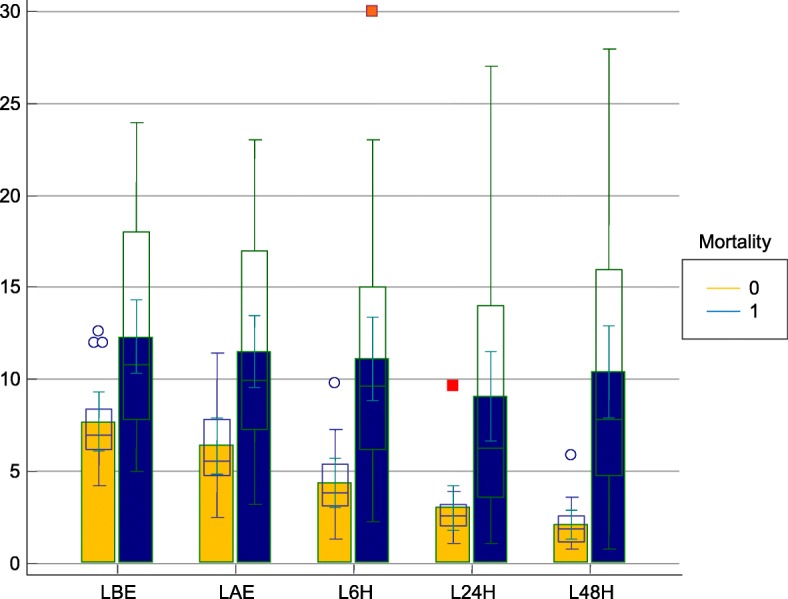
Changes in blood lactate levels detected on different time points depending on mortality. Mortality 1, represents the nonthe survivor group while mortality 0 represents the survivor group. LBE, Lactate level pre-ECMO (mmol/l); LAE, Lactate level post-ECMO (mmol/l); L6 h, Lactate level 6 h after ECMO implantation; L24 h, Lactate level 24 h after ECMO implantation; L48 h, Lactate level 48 h after ECMO implantation

Lactate levels were elevated before ECMO implantation (10.9 ± 5.4 mmol/l) and there was a significant difference depending on 30-day mortality (the survivors vs. non-the survivors: 7.6 ± 2.7 mmol/l vs 12.3 ± 5.7 mmol/l; *p* = 0.008). All patients presented a 10% decrease in basal lactate levels but the duration of this period was significantly different between groups. Even the lactate value 1 h after ECMO support was different statistically and predictive concerning 30-day mortality (the survivors vs. non-the survivors: 6.4 ± 2.6 mmol/l vs 11.5 ± 5.6 mmol/l; *p* = 0.003). However, the significance of the lactate difference considering post-ECMO and pre-ECMO lactate values was more prominent (− 1.2 ± 0.6 vs − 0.9 ± 0.8; *p* = 0.002). Thus, we decided to make assumptions with lactate differences (LC-1, and LC-2) rather than the absolute lactate value. Nevertheless, we summarize lactate values and LC-2 in Fig. 2 and compare them according to the mortality factor. It was obvious that the difference in LC-2 was the most remarkable one among the variables.

**Fig. 3:**
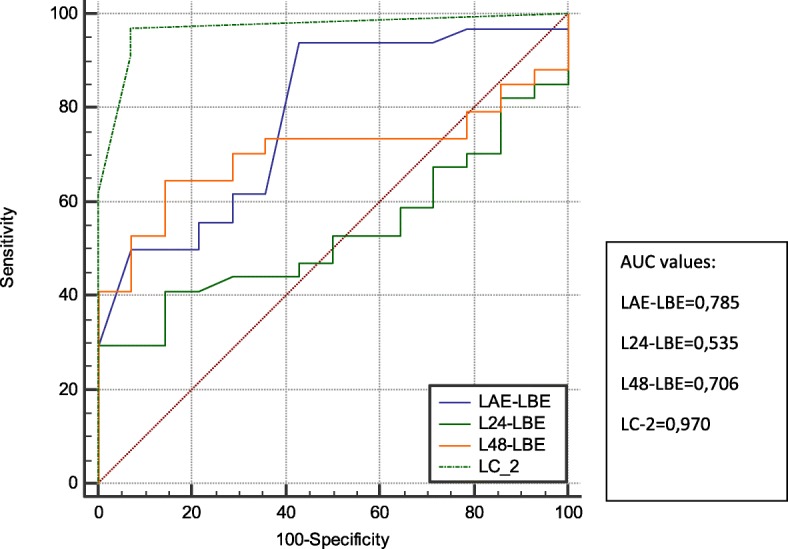
ROC curves to demonstrate the power of predictively of LC-1 at each time point and LC-2 on 30-day mortality. ROC, receiver operating characteristics; AUC, area under the (ROC) curve; LBE, Lactate level pre-ECMO (mmol/l); LAE, Lactate level post-ECMO (mmol/l); L24 h, Lactate level 24 h after ECMO implantation; L48 h, Lactate level 48 h after ECMO implantation; LC-2, duration between ECMO implantation time and time point that Lactate cleared more than 10% of the initial level

**Fig. 4:**
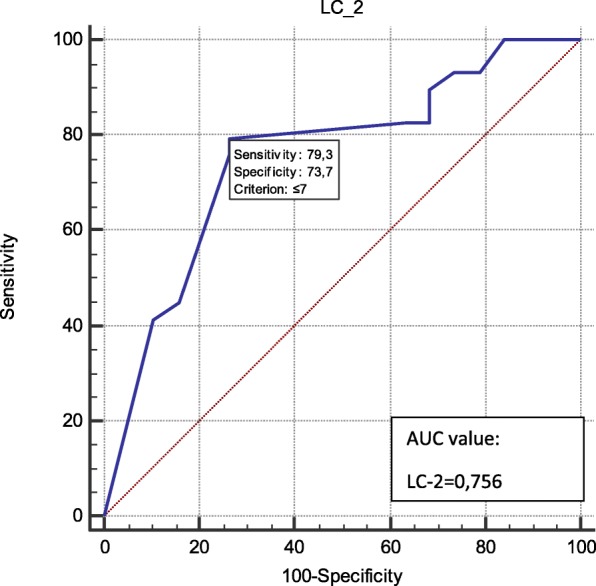
ROC curve to demonstrate the predictive value of lactate clearance-2 on the length of stay in ICU more than 5 days. ROC, receiver operating characteristics; AUC, area under the (ROC) curve; LC-2, the duration between ECMO implantation time and time point that Lactate cleared more than 10% of the initial level

In Table 3, lactate levels, LC-1 at specific time points and LC-2 were compared depending on 30-day mortality. In addition, the predictive value of lactate level, LC-1 for specific time points and LC-2 were assessed by using ROC curve analysis (Fig. 3). It is clearly seen that LC-2 (as the duration for lactate clearance of more than 10% of initial level) had a stronger predictive power. AUC ROC values with 95% CI were 0.97 for LC-2, 0.706 for LC-1 (48 h), and 0.785 for LC-1 (1 h) respectively. Apart from this, LC-2 was related to and had predictive power with LOS in ICU more than 5 days (Fig. 4).

**Table 3:** Lactate levels, lactate clearance-1 at specific time points and lactate clearance-2 were compared

	All (*n* = 48)	Survivors (*n* = 14)	Non-survivors (*n* = 34)	*p*-value^*^
LL pre-ECMO (mmol/l)	10.9 ± 5.4	7.6 ± 2.7	12.3 ± 5.7	0.008
LL post-ECMO (mmol/l)	10.0 ± 5.4	6.4 ± 2.6	11.5 ± 5.6	0.003
LL postECMO- LL preECMO (mmol/l)	−0.9 ± 0.8	−1.2 ± 0.6	−0.9 ± 0.8	0.002
LC-1 6 h (mmol/l)	−1.8 ± 4.4	−3.2 ± 1.1	− 1.8 ± 4.4	0.017
LC-1 24 h (mmol/l)	−3.6 ± 5.6	−4.6 ± 2.2	− 3.6 ± 5.6	0.634
LC-1 48 h (mmol/l)	−2.9 ± 6.2	−5.5 ± 2.6	−2.9 ± 6.2	0.023
LC-2 (hours)	7.0 ± 6.1	1.3 ± 1.3	9.4 ± 5.8	< 0.001

Subgroups (The survivors versus Non-the survivors) refer to 30-day mortality. *p*-values calculated for comparison of the survivors versus non-the survivors by statistical analysis

Data are mean ± SD or *n* (%)

^*^Determined by Mann-Whitney U test or Spearman’s rho test

*Abbreviations*: *ECMO* extracorporeal membrane oxygenation, *LL* lactate level at specific time, *LC-1* lactate clearance at specific time, *LC-2* the duration between ECMO implantation time and time point that Lactate cleared more than 10% of the initial level

## Discussion

ECMO is claimed to be a very effective tool for providing and advancing systemic circulation and provide gas exchange. In cardiogenic shock and cardiac arrest, it is used as a mechanical support mostly. The goal of the ECMO usage is myocardial rest while protecting end-organ perfusion and supporting good neurologic function after the course [1]. The mortality rate is reported between 60 and 80% [10] and our 30-day mortality rate in cardiogenic shock with ECMO support is similar (70.8%).

The imbalance between production and clearance of lactate causes higher lactate levels and LC may prognosticate the mortality in patients on extracorporeal circulatory support. It has also been correlated with survival, in post-cardiac arrest, after the return of spontaneous circulation with the help of ECMO [6]. Li et al. [5] noticed that fluctuations in lactate values following the ECMO implantation can presage in-hospital mortality in post-cardiac surgery patients. Hence the improvement in LC and the decreasing trend of lactate levels may be considered as a sign of improvement in perfusion and oxygenation during eCPR, in patients with refractory cardiac arrest on ECMO support [5]. Serial lactate measurements over time or lactate clearance have been reported to be clinically more reliable than absolute values of lactate, as a surrogate for the magnitude and duration of global tissue hypoxia, for risk stratification in different pathologic conditions ranging from sepsis to trauma [1, 6, 11].

In our patients, we measured lactate levels and calculated LC-1 at 6, 24 and 48 h after initiation of ECMO. We also measured LC-2 (time) as defined in the material method section. LC-1 at 48 h and LC-2 were found significantly related to mortality while LC-1 at 6 and 24 h had no relation. Especially, the LC-2 correlation was significant statistically with greater strength (*p* < 0.001). With AUC calculation, LC-2 has a strong discrimination (AUC = 0.97) on 30-day the survivors and nonthe survivors. Lactate level post-ECMO (mmol/l) (LAE) - Lactate level pre-ECMO (mmol/l) (LBE) (AUC = 0.785), Lactate level 48 h after ECMO implantation (L48 h) -LBE (AUC = 0.706) showed moderate predictive power on 30-day mortality. Due to the fact that in 13 patients, ECMO support could not achieve to decrease lactate value below the level of 4 mmol/l, this cutoff value as a variable could not be used for mortality prediction model in this study. This lactate level is important because it is claimed as a predictor of death in some studies [12, 13].

Even in some studies, it is postulated that ECMO decannulation is proper when the LC remained within 10% of baseline [6, 14]. However, our study was designed in a retrospective manner so we could only check LC for mortality prediction and correlation. It is clearly shown that the dynamic changes of lactate levels are related to mortality and LOS in ICU more than 5 days.

In the present study, lactate levels were elevated before ECMO implantation and there was a significant difference depending on 30-day mortality (*p* = 0.008). This correlation implies that early support by ECMO probably avoids progression to multiorgan failure by improving tissue perfusion.

### Limitations

There are some limitations related to this study that need to be acknowledged. First, the retrospective nature is a major limitation of this analysis. Secondly, the small sample size and limited data are restricting our conclusions. Although different persons did data acquisition and statistical evaluation to overcome bias effect, one may hesitate about it.

## Conclusion

A sufficient life support is a must for successful treatment of life-threatening situations and dynamic lactate indices empower evaluation of circulatory support by ECMO. Based on our results, an early insertion of ECMO before lactate gets high was suggested. Serial changes on lactate levels and calculation of its clearance may be superior to single lactate value on both effective circulatory support and as prognostic prediction marker. LC-2 showed a strong discrimination on 30-day mortality. Furthermore observing the course of blood lactate during ECMO therapy is a valuable tool not only for prediction of mortality but also for LOS in ICU.

## Abbreviations

AUC: Area Under Curve
ECMO: Extracorporeal membrane oxygenation
eCPR: extracorporeal cardiopulmonary resuscitation
ICU: Intensive care unit
L48 h: Lactate level 48 h after ECMO implantation
LAE: Lactate level post-ECMO (mmol/l)
LBE: Lactate level pre-ECMO (mmol/l)
LC-1: Lactate clearance-1
LC-2: Lactate clearance-2
LOS: Length of stay
LVAD: Left ventricular assist device
ROC: Receiver operating characteristic
ScvO_2_: Central venous oxygen saturation
SD: Standard deviation
V-A ECMO: Veno-arterial-ECMO
V-V ECMO: Veno-venous ECMO
